# Performance Improvement of Gold Electrode towards Methanol Electrooxidation in Akaline Medium: Enhanced Current Density Achieved with Poly(aniline-*co*-2-hydroxyaniline) Coating at Low Overpotential

**DOI:** 10.3390/polym14020305

**Published:** 2022-01-13

**Authors:** Anwar ul Haq Ali Shah, Sadaf Zia, Gul Rahman, Salma Bilal

**Affiliations:** 1 Institute of Chemical Sciences, University of Peshawar, Peshawar 25120, Pakistan; sadafzia789@gmail.com (S.Z.); gul_rahman47@uop.edu.pk (G.R.); 2 National Center of Excellence in Physical Chemistry, University of Peshawar, Peshawar 25120, Pakistan

**Keywords:** poly(aniline-*co*-2-hydroxyaniline), electrochemistry, electrooxidation, alkaline fuel cell

## Abstract

Electronically conducting poly (aniline-*co*-2-hydroxyaniline) (PACHA), a copolymer of aniline and 2-hydroxyaniline (2HA), was electrochemically coated on gold substrate for methanol electrooxidation in alkaline media. The electrochemical behavior of PACHA coated gold electrode towards methanol electrooxidation was investigated via cyclic voltammetry (CV) and electrochemical impedance spectroscopy (EIS) for application in an alkaline fuel cell. Methanol electrooxidation was observed at two different electrode potentials depending on the concentration of the base. At the PACHA coated gold electrode, the methanol oxidation peak was observed at lower overpotential (at 0.19 V) in a solution of high base concentration (1.8 M NaOH), which was 30 mV lower than the peak for the uncoated gold electrode. In addition, the Faradic current Imax obtained on the PACHA coated electrode (20 mA) was two times higher as compared to the Faradic current Imax of the un-modified gold electrode (10 mA). In solution of lower base concentration (0.06 M NaOH), the electrooxidation of methanol became sluggish on both electrodes, as indicated by peak shifting towards positive potential and with reduced faradaic current (at 0.74 V on PACHA coated electrode; Imax 10 mA). The electrooxidation of methanol at both lower and higher electrode potentials was analyzed mechanistically and discussed in light of the literature. EIS results were interpreted using Nyquist and Bode plots. The charge transfer resistance was decreased and pseudo-capacitive behavior changed to conductive behavior when external applied potential was increased from 0.1 V to 0.4 V.

## 1. Introduction

Fuel cells are currently in high demand as energy fuel sources because of effective use of fossil fuels and reduction of air pollutants throughout the world. Various kinds of fuel cells have been designed up until now, including molten carbonate fuel cells, hydrogen fuel cells (also called proton-exchange membrane fuel cells), direct methanol fuel cells (DMFCs), polymer electrolytic membrane fuel cells, alkaline fuel cells, and phosphoric acid fuel cells [1]. DMFCs are currently considered as the best energy producing cells because of higher energy density, simplicity, safety, eco-friendliness, and low emission of pollutants [2,3,4]. Moreover, in terms of storage and portability, DMFCs are considered more favorable as compared to hydrogen fuel cell [5].

In DMFCs, methanol electrooxidation has extensively been studied both in acidic and alkaline media. However, electrooxidation of methanol in acidic media is associated with disadvantages like poor electrode kinetics and ohmic polarization of the desired system under study [6,7], lower feasibility of an anodic reaction, fragile catalytic activity [8], and fuel wastage due to high methanol crossover. Furthermore, due to their low temperature operations, acidic fuel cells exhibit CO poisoning of the catalyst as well [9]. In contrast, the study of alkaline fuel cells is quite worthy and has attained major attention around the globe. For the first time since 1955, DMFCs were studied under alkaline media by employing porous Ni for oxidation reaction and porous Ni-Ag catalyst for reduction purposes [10]. The current passage of electrons in basic fuel cells is due to the transfer of OH^−^ ions. Anodic electrooxidation of CH_3_OH in basic media was reported to be more practical than in acidic media [6]. It is notable that alkaline fuel cells are more distinct owing to their remarkable electrode kinetics of O_2_ reduction compared to acidic fuel cells [11].

When considering an alkaline medium for methanol oxidation, one of the challenging tasks is the choice of the catalyst. It has been reported that electrooxidation of methanol in alkaline media attains structure sensitivity [10], which has made a way for utilizing precious metals [12], other than platinum and platinum-modified catalysts, like Ni, Pd, Ag, Cu@NiCo, and some kinds of perovskite oxide catalysts [13,14,15,16,17]. Some recent report shows efficient iron based catalyst [18] and palladium based catalyst mixed with rhodium, which showed higher faradic current densities for methanol electrooxidation than Pt and Pt-based catalysts in alkaline fuel cells [19]. Li et al. fabricated nano-composite membranes designated as chitosan nano-particles integrated into a quaternized poly (vinylalcohol) matrix that are capable of high ionic conductivity and high power density for alkaline fuel cells [20]. Kruusinberg et al. have reported metal free nitrogen doped carbon nanotubes leached in alkaline media, which were found to be very efficient in regard to oxygen reduction reactions and could successfully be employed in alkaline fuel cells [21]. Galvan et al. have recently highlighted the importance of ionomer in the design of alkaline direct methanol fuel cells for high power generation [22].

For alkaline media, the most effectively practiced substrate is gold [8,23,24]. The catalytic activity of gold in alkaline medium was highly pronounced for numerous organic species [8]. Electrocatalytic oxidation of methanol by gold was reported to be enhanced by CO in alkaline media [25]. Hernandaz et al. observed unusual electrocatalytic activity of gold nanoparticles in alkaline media towards methanol oxidation [26]. Parpot et al. investigated the pH effect on the electrooxidation of various readily available organic species in alkaline media [27]. Cherevko et al. studied electro catalytic oxidation of methanol on coupled Au modified electrodes (i.e., Au/Pt and Au/Pd) and reported on high electrocatalytic activity of coupled Au modified electrodes in alkaline media [28]. Zhang et al. studied electrooxidation of alcohols on Au and Pt substrates by various electrochemical techniques and reported Au as the most efficient catalyst in alkaline media [29].

Enhanced electrocatalytic activity of Ni impregnated polyaniline/poly (2-hydroxyaniline) bilayer structure coated Pt substrate towards electrooxidation of methanol in alkaline media was reported [30]. Poly(aniline-*co*-2-hydroxyaniline) (PACHA), a copolymer of aniline and 2-hydroxyaniline (2HA) has been studied extensively [31,32,33]. It has shown promising electrochemical activity and good redox response in neutral and even basic media [34]. This is because of the ability of this copolymer to protonate internally, proton provided by –OH group, in neutral and basic media. Because of these advantages it was applied in the fields of sensors [29,35] and Zn-rechargeable batteries [28]. PACHA coated gold sheet electrode was recently reported for electrooxidation of ascorbic acid in citrate [36]. To the best of our knowledge, this fascinating derivative of polyaniline has not been tested in the electrooxidation of methanol in either acidic or basic media. It seems very interesting to test PACHA coated electrodes for the electrooxidation of methanol, especially in alkaline media, because of its electrochemical activity. In this article we report preliminary results of electrocatalytic oxidation of methanol on PACHA coated gold disc electrodes in alkaline media. The electrooxidation of methanol was analyzed over a wide range of sodium hydroxide (NaOH) concentrations via cyclic voltammetry (CV) and electrochemical impedance spectroscopy (EIS). The behavior of PACHA coated electrode towards methanol was compared with polyaniline and poly(2-hydroxyaniline) (PHA) coated Au substrate in the same media.

## 2. Experimental

### 2.1. Materials and Equipment

Reagent grade aniline (Riedel-de haen) was received and distilled under vacuum. The resulting transparent aniline solution was stored in a nitrogen atmosphere. Reagent grade 2-hydroxyaniline (ACROS), methanol (Riedel-de haen), sulphuric acid (Riedel-de haen), sodium hydroxide (NaOH), and other reagent grade chemicals were used. For electrochemical measurements and EIS study, a Gamry Reference 600 Potentiostate/Galvanostate (Warminster, PA, USA) was used. Gold disk (2 mm diameter) (ALS Japan) and gold wire electrodes were employed as working and counter electrodes, respectively. In all electrochemical measurements, saturated calomel electrode (SCE) was used as reference electrode. The FTIR spectra were recorded with IRAffinity-1S Fourier transform infrared spectrophotometer (Shimadzo, Japan) with ATR accessory in transmittance mode. DC conductivity measurements were carried out with four probe conductivity setup (Jandel Engineering Limited, Leighton Buzzard, Beds, UK) on press pellets.

### 2.2. Preparation of Copolymer Coated Electrode and Electrochemical Characterization

Poly(aniline-*co*-2-hydroxyaniline) coated Au electrode was prepared electrochemically by depositing the copolymer on the electrode surface from a mixture of 20 m**M** aniline and 1 m**M** 2-hydroxyaniline in 0.5 **M** sulphuric acid solution. The copolymer was deposited potentiodynamically, by cycling the potential from −0.20 to 1.10 V at a scan rate of 50 mV/s. Polyaniline and poly(2-hydroxyaniline) were prepared electrochemically at the same scan rate. The freshly prepared electrode was rinsed with distilled water several times to remove unreacted species. The electrochemical activity of PACHA coated and uncoated gold electrodes towards methanol oxidation were measured by cyclic voltammetry and electrochemical impedance spectroscopy. The behavior of PACHA coated electrode towards methanol electrooxidation was optimized by recording CVs in methanol solutions of different concentration in alkaline medium. The effect of scan rate, ranging from 25 to 200 mV/s, on the electrooxidation of methanol was studied by recording CVs of the optimized methanol concentration in alkaline medium.

### 2.3. Sample Preparation for FTIR Analysis

Polyaniline and Poly(aniline-*co*-2-hydroxyaniline) were prepared electrochemically on the gold electrode as described in Section 2.2. After washing with plenty of water, PANI and PACHA were scratched from the electrode surface, dried in an oven at 60 °C for several hours, and ground down into power form. The film of poly(2-hydroxyaniline) with was so thin that it could not be scratched from the electrode surface.

## 3. Results and Discussion

### 3.1. Electrochemical Preparation of Poly(aniline-co-2-hydroxyaniline) Coated Gold Electrode and Characterization

**Figure 1:**
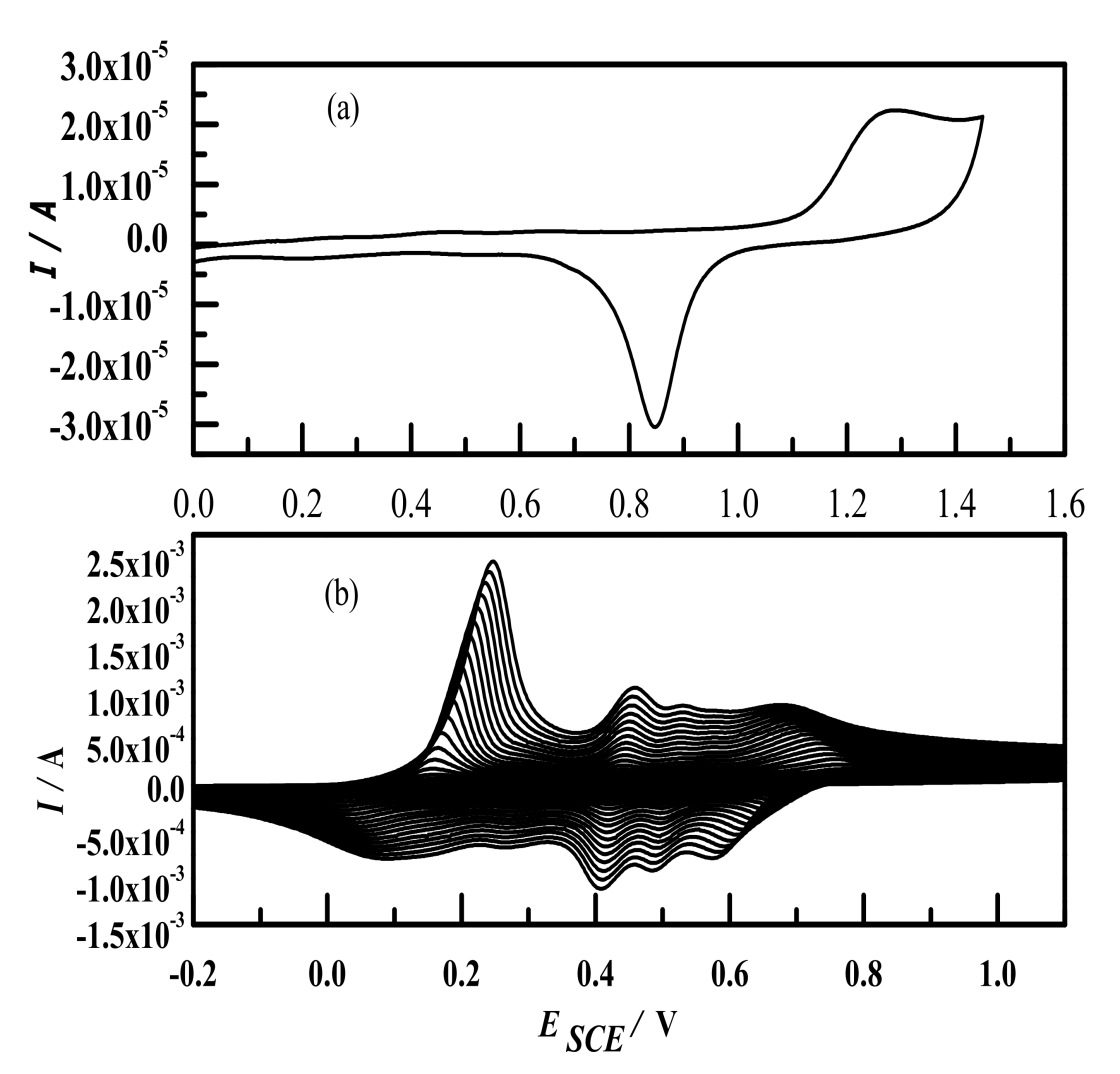
(**a**) Cyclic voltammogram of gold disc electrode recorded in 0.5 **M** sulphuric acid solution at 50 mV/s. (**b**) Cyclic voltammogram of gold disc electrode recorded during electrolysis of a solution containing 1 m**M** 2-hydroxyaniline and 20 m**M** aniline in 0.5 **M** sulphuric acid solution at 50 mV/s.

Figure 1a shows a cyclic voltammogram of bare gold disc electrode in 0.5 **M** H_2_SO_4_ solution. It displays characteristic oxidation and reduction peaks, as is well documented in the already reported literature. The CV curves recorded with the oxidation of monomers of aniline, 2-hydroxyaniline, and their mixture are shown in Figure S1. There is only one oxidation peak observed at 1.0 V on the forward scan of the CV curve recorded in aniline solution corresponding to the oxidation of amino group of aniline monomers (Figure S1a) in accordance with the previously reported data [37,38]. Two oxidation peaks at 0.67 V and 0.95 V are observed on the forward scan of the CV curve recorded in 2-hydroxyaniline solution (Figure S1b). The first peak at 0.67 V arises due to the oxidation of –OH group because phenols give an oxidation peak around this potential, as is well documented in several reports [39,40]. The second peak at 0.95 V is due to oxidation of the amino group of 2-hydroxaniline. This peak is slightly shifted towards less positive potential (by 50 mV) as compared to the peak observed in the CV curve of aniline oxidation. This shift is attributed to the increase in electron density of the conjugated bond system due to introduction of an electron donating hydroxyl substituent. The CV curve (Figure S1c) recorded in the mixture of aniline and 2-hydroxyaniline solution reflects different behavior. Two anodic and three cathodic peaks are observed in the CV curve for the first cycle. The anodic peak at lower potential (0.67 V) is due to oxidation of the –OH group of 2-hydroxyaniline, whereas the other peak at higher potential (1.0 V) is assigned to oxidation of amino groups both from aniline and 2-hydroxyaniline, causing copolymerization. The peak at 1.0 V is very intense as compared to the peak at 0.67 V. This is attributed to the greater number of amino groups available for oxidation from both the aniline and 2-hydroxyaniline monomers as compared to the number of –OH groups. This explanation is quite reasonable because the intensity of both these peaks is the same in Figure S1b, in which the number of –OH and –NH_2_ are equal in 2-hydroxyaniline solution.

In the CV curve of the second cycle (Figure 1b), three anodic peaks with a shoulder and four cathodic peaks were observed. Beyond the fifth cycle, four redox peaks were observed in the CV curves recorded in the mixed solution of aniline and 2-hydroxyaniline. The oxidation and reduction peaks around 0.32/0.28 V are not observed in the electrolysis of solution containing only aniline. CV curves show three redox couples during polymerization of aniline. The additional pair of oxidation and reduction peaks around 0.32/0.28 V in Figure 1b arises from the copolymer PACHA deposited on the electrode surface, as discussed comprehensively in previous reports [27,28,29,30].

**Scheme 1:**
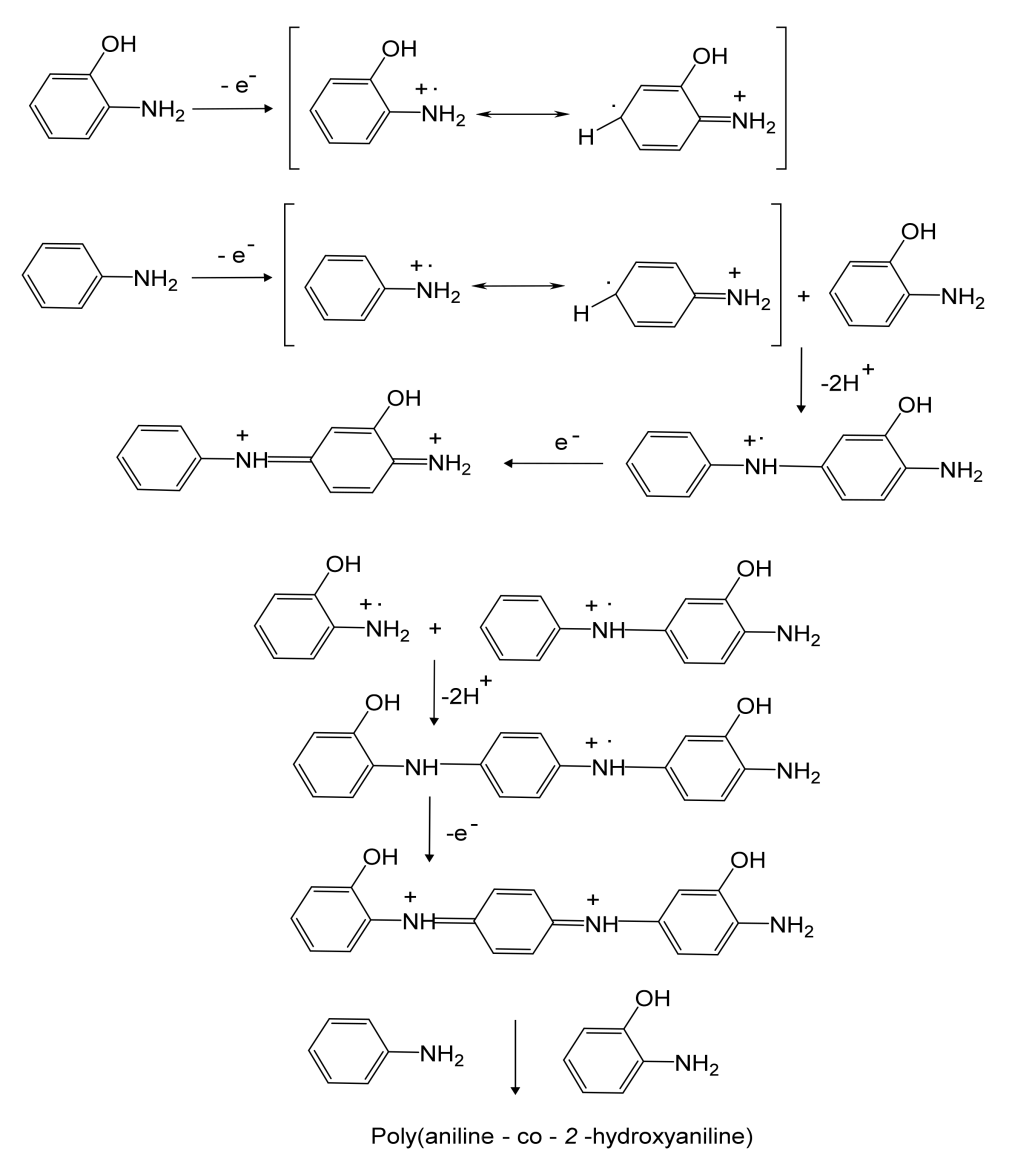
Copolymerization of aniline and 2-hydroxyaniline.

The mixing of 2-hydroxyaniline into aniline not only influences the electrochemical features of polymer deposition on the electrode surface but also decreases greatly the rate. This is obvious because the degree of incorporation of each co-monomer into the copolymer depends on the reactivity ratio and copolymerization kinetics. The structure and properties of polymers also depend on the synthetic conditions, e.g., the concentration of monomer, concentration of electrolyte, mode of electrosynthesis (i.e., potential cycling, galvanostatic, and potentiostatic), and potential of synthesis, etc. The composition of copolymer formed can be discussed by considering monomer concentration and their reactivities during the copolymerization process in light of Mayo–Lewis and other related theories, but they often lead to erroneous results, as reported recently with computational modeling [41]. The sequence of copolymerization was systemically followed by in situ UV-Vis and Raman spectroelectrochemical measurements [42], where head to tail coupling of co-monomers was reported according to the following Scheme 1. This is reconsidered here again on the desire of a reviewer.

**Figure 2:**
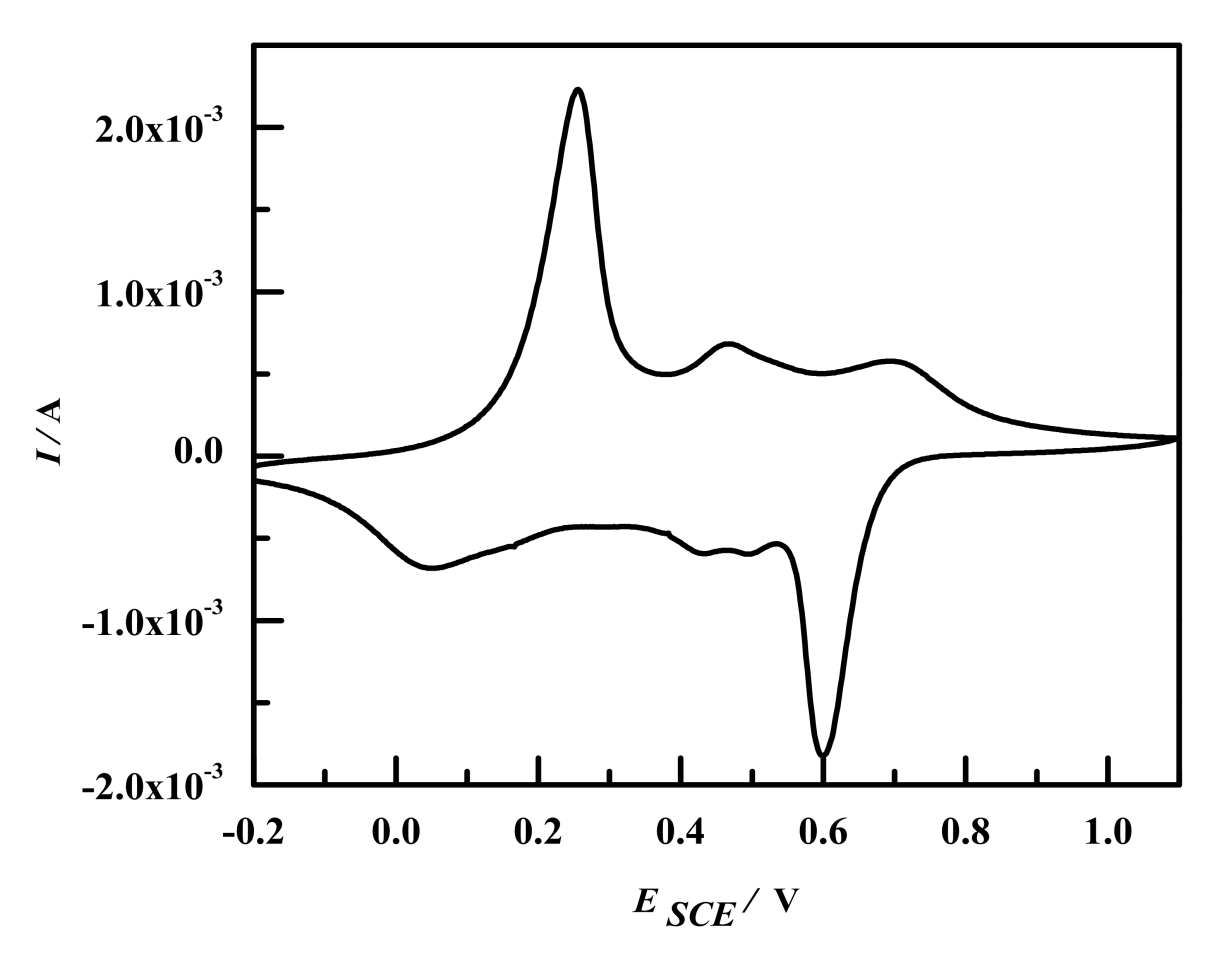
Cyclic voltammogram of PACHA coated gold electrode in 0.5 **M** sulphuric acid solution at 50 mV/s.

Figure 2 shows the CV curve of a PACHA coated electrode in monomer free electrolyte solution. It displays polyaniline-like electrochemical features as displayed in Figure S2 where two redox processes are clearly observed, as reported elsewhere [37,38]. The anodic peak around 0.26 V corresponds to the transformation of the leucoemeraldine form of the polymer to an emeraldine state, whereas the peak at 0.70 V corresponds to further transformation of the emeraldine form to a pernigraniline state. In addition to these two anodic peaks, there is a third anodic peak at 0.47 V. This anodic peak is associated with two cathodic peaks on the backward scan at 0.43 V and 0.49 V. The appearance of extra peaks around 0.47–0.49 V indicate the deposition of material different from that of polyaniline. The redox peaks in this region may arise either from the oxidation and reduction of poly(2-hydroxyaniline) on polyaniline film or from the PACHA itself. However, poly(2-hydroxyaniline) does not give redox peaks in this region. This clearly indicates that a copolymer has been deposited on the electrode surface.

**Figure 3:**
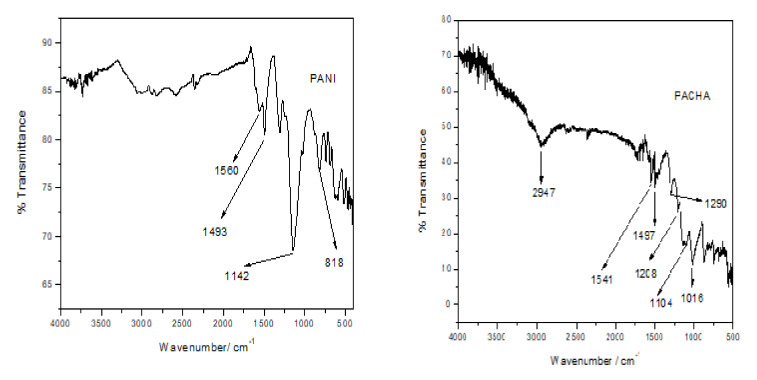
FTIR spectra of PANI and PACHA.

Further characterization of PACHA was done with FTIR spectral analysis. Figure 3 shows the FTIR spectrum of PACHA in comparison with the PANI spectrum.

Major bands in both spectra are labeled with corresponding frequencies in wavenumbers. The C=N and C=C stretching vibrations of the quinoid diamine unit of the polymer results in bands at 1541 cm^−1^ [43], whereas C=C ring stretching of the diamine benzoid unit results in bands in the region of 1487 cm^−1^ [44]. These bands are observed at 1560 and 1493 cm^−1^, respectively, in the PANI spectrum. The C-N stretching of the secondary aromatic amine is supported by the presence of the band at 1290 cm^−1^ [45]. Similarly, out of plane bending modes of aromatic C-H are depicted by the presence of a band at 798 cm^−1^. The peaks between 3000–3500 cm^−1^ are due to vibrational bands of N-H groups [46,47]. There are bands at 2947, 1208, and 1016 cm^−1^ in the FTIR spectrum of PACHA. However, one cannot find such bands in the PANI spectrum. The appearance of these bands in the FTIR spectrum of PACHA gives a clue of the incorporation of 2-hydroxyaniline units in the polymer backbone because the FTIR spectrum of 2-hydroaniline also display bands at 1031–1200 cm^−1^ and are attributed to C–O stretching vibrations [48].The band at 2947 cm^−1^ is attributed to C–O–H deformation vibration, as this band is also observed in the FTIR spectra of phenols and 2-hydroxyaniline [48,49]. The appearance of a band at 2947 cm^−1^ in the PACHA spectrum strongly supports copolymer formation. The issue of copolymerization was very nicely discussed in [50], where other techniques such as conductivity measurements and solubility tests have been reviewed in addition to vibrational spectroscopies. Indeed, the conductivity of PACHA (0.25 S/cm) was lower as compared to PANI. This decrease in conductivity is expected due to the presence of side –OH groups, which cause separation in polymer chains. It is useful to mention here that the electrochemically synthesized PANI, poly(2-hydroxyaniline), and PACHA in acidic medium are not soluble in common solvents, therefore, the solubility test cannot be performed as reported elsewhere [51].

### 3.2. Electro Oxidation of Methanol on PACHA Coated Gold

Electrooxidation of methanol in alkaline media has been studied by many researchers. It is interesting to note that methanol gets oxidized in different potential regions on gold substrate in alkaline media. Some researchers have reported methanol oxidation in lower potential regions [21,52,53], whereas others have reported it in the higher potential regions [26]. It is worthwhile to note that these literatures report the electrooxidation of methanol on the Au electrode in alkaline media in two different regions independently. Therefore, both lower and higher potential regions were selected with the aim to study the behavior of PACHA coated Au electrode towards methanol oxidation in alkaline media and compare the results with uncoated Au electrode.

(a) Electro-oxidation of Methanol at Lower Potential Region

**Figure 4:**
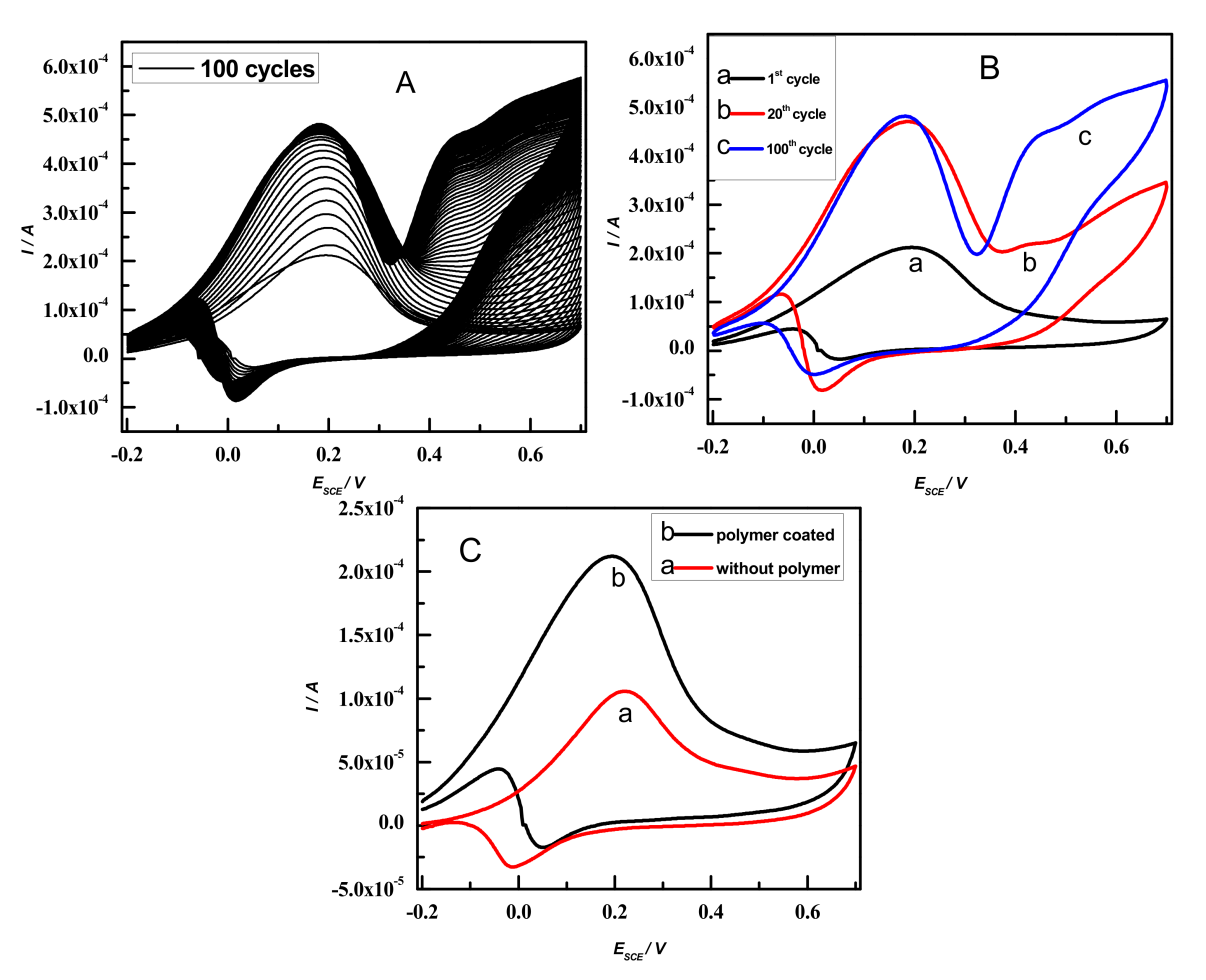
Cyclic voltammograms of PACHA coated gold substrate in 1.8 **M** NaOH + 5 **M** CH_3_OH solution at 50 mV/s. (**A**) 100 cycles; (**B**) 1st, 20th, and 100th cycles; (**C**) CV curves of methanol electro oxidation recorded on PACHA coated and uncoated gold electrodes.

Figure 4A shows cyclic voltammograms recorded with electro oxidation of methanol on PACHA coated gold electrodes in the potential region of E*_SCE_*= −0.20 V to E*_SCE_*= 0.70 V at a scan rate of 50 mV s^−1^. In the very first potential sweep, a broad anodic peak is observed at 0.19 V, indicating strong adsorption of OH^-^ anions on the PACHA coated electrode surface [33,54]. This might be attributed to the porous texture of PACHA deposited on the Au substrate with vacant spaces. It is assumed that the OH^-^ anions get insight to the vacant spaces and form an oxide layer, causing the formation of “pre-oxidation species” [55]. The faradic current of the very first sweep is 0.212 mA. The increase in faradic current with continuous subsequent sweeping clearly indicates more chemisorption of OH^-^ anions on the available surface [35]. In the methanol electro-oxidation process, these OH^-^ anions are assumed to act as electron transfer mediators [56]. The increase in anodic peak current was observed up to the 20th cycle, with maximum current around 0.471 mA. Beyond the 20th cycle, no incremental increase in faradic current was observed, indicating that no more OH^-^ anions get adsorbed on the surface, because of the unavailability of the vacancies. Figure 4B shows the CV curves of the 1st, 20th, and 100th cycles of Figure 4A. The same peak current of the 20th and 100th cycles clearly show saturation of the surface with OH^-^ anions at the 20th cycle.

Electro-oxidation of methanol in the lower potential region is attributed to methanol oxidation to formats by losing four of its electrons according to Equation (1) [35].

CH_3_OH + 5OH^−^ ↔ HCOO^−^ + 4H_2_O + 4e^−^ (1)

Furthermore, it was also reported that at high pH value lower potential intermediates like formaldehyde and formic acid are formed. Elsewhere the CH_3_O^−^ anions at high pH (pH = 11) form as reactive specie, and increase in its concentration with pH increase was reported [22]. The formation of CH_3_O^−^ anions at higher pH values was based on deprotonation of methanol according to Equation (2):

CH_3_OH ↔ CH_3_O^−^ + H^+^ (2)

Figure 4C shows CVs curves of electro-oxidation of methanol on PACHA coated and uncoated gold electrodes. It is clear from the figure that on bare gold electrodes, the methanol oxidation peak appears at 0.22 V, whereas on PACHA coated electrodes, the peak originates at 0.19 V. Similarly, the anodic peak current is enhanced two times on PACHA coated electrode as compared to on bare gold. These results show the high electro-catalytic activity of PACHA towards methanol electro-oxidation. This could be attributed to the high electrochemically active available area and easy charge transfer at the polymer and electrolyte interface.

**Figure 5:**
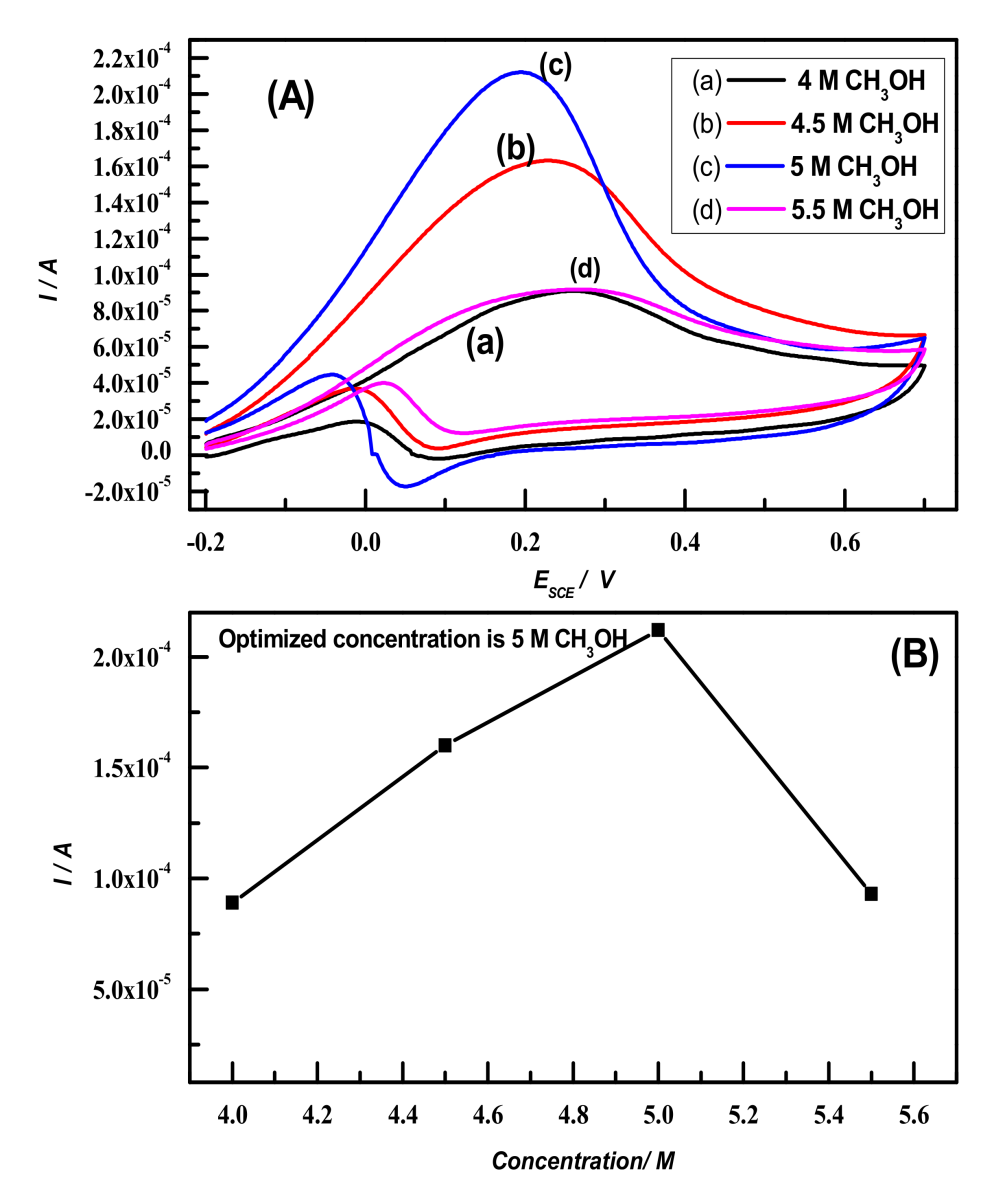
(**A**) CVs of PACHA coated electrodes recoded in different concentrations of methanol (as indicated) in 1.8 M NaOH at 50 mV/s; (**B**) effect of methanol concentration on anodic peak current density in 1.8 M NaOH.

The effect of methanol concentration on anodic current density was investigated by recording CVs of PACHA coated electrodes in 1.8 **M** NaOH containing different concentration of methanol, as depicted in Figure 5A. This set of experiments shows that anodic current density increases with the increase in methanol concentration but up to certain limit, i.e., 5 **M** CH_3_OH concentration where we can elucidate that point as a saturation point where no more active sites are available for methanol oxidation reactions. Beyond 5 **M** concentration, a decrease in anodic current density can be observed, as clearly shown in Figure 5B. Therefore 5 **M** could be considered as the optimal concentration of methanol in the current investigation [57]. When studying electrooxidation of methanol in basic media, it is assumed that hydroxyl ions, methanol, and gold are the responsible species for the oxidation process. During the methanol oxidation reaction in basic media, hydroxyl ions produce abundantly, which are strongly adsorbed by the gold electrode surface by producing numerous gold oxide ions, which then make way for methanol oxidation [36]. It is also observed that there is continuous peak shift towards negative potential with the increase in methanol concentration, which means that oxidation of methanol on PACHA coated gold electrodes is a diffusion controlled phenomenon [26,58] or may also be due to oxide layer formation [36]. Elsewhere electrooxidation of 1 **M** methanol in 0.5 **M** NaOH was reported around −0.1–0.2 V on pladium-polyaniline coated gold electrodes [52].

**Figure 6:**
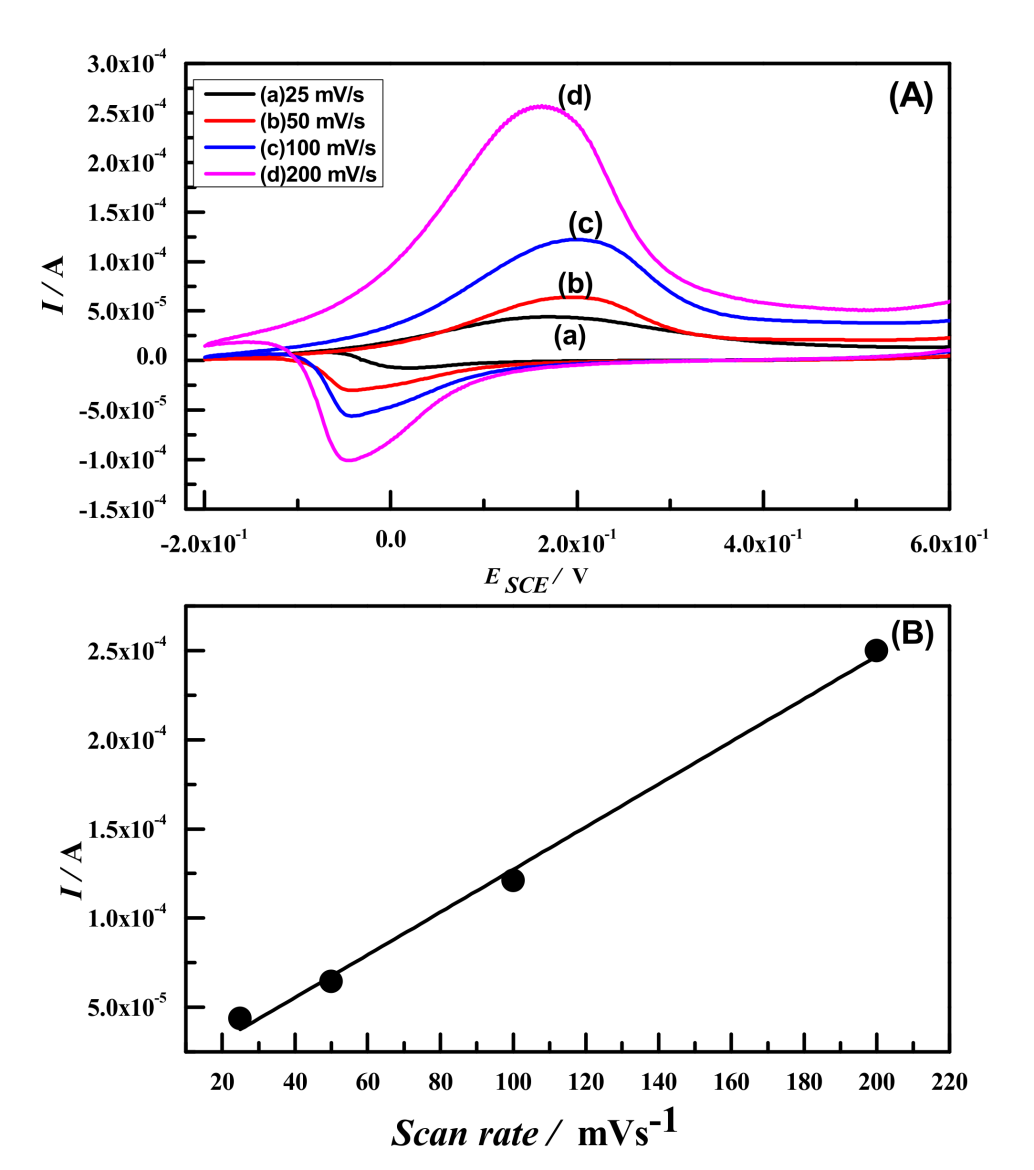
(**A**) CV curves of PACHA coated gold electrode recorded in 1.8 **M** NaOH + 5 **M** CH_3_OH at 25, 50, 100, and 200 mV/s; (**B**) plot of anodic peak current versus scan rate.

The electro-catalytic behavior of PACHA coated electrodes towards methanol oxidation at different scan rates was studied in the same basic media, i.e., in 1.8 M NaOH at optimum methanol concentration (5 **M**), as shown in Figure 6A. The anodic peak current was found to increase with the increase in scan rate from 25 mV/s to 200 mV/s, indicating that the methanol oxidation reaction at the PACHA coated electrode attained good stability with the increase in scan rate [59]. A slight shift in peak potentials is observed with the increase in scan rate, indicating contribution from electrochemical polarization and kinetics constraints of methanol oxidation on PACHA modified gold electrodes [38,60,61]. The electrooxidation of species at the electrode surfaces are either adsorption controlled or diffusion controlled. The anodic peak current is proportional to sweep rate in the studied range of 25–200 mV/s, as shown in Figure 6B. The concentration of redox species on the surface of the PACHA coated electrode and active surface area of the coated electrode can be estimated from the slope of anodic current vs. scan rate plot [62,63].

Alternatively, using the Randles–Sevick equation diffusion coefficient can be calculated from slope of anodic peak current versus square root of the seep rate. This approach has been discussed previously for investigation of OH^−^ diffusion capability on electrode materials during methanol oxidation [64]. The contributions of the diffusion component of the capacitance of hollandite with carbon coating were recently discussed from the graph of anodic current versus square root of scan rate [65]. In the present study, the plot of anodic peak current versus square root of scan rate is not linear, as shown in Figure S3 in the supporting information. This indicates that the methanol oxidation on PACHA coated electrodes is less controlled by diffusion, and the major contribution arises from the adsorption of OH^−^ into the porous texture of PACHA.

(b) Electrooxidation of Methanol at Higher Potential Region

**Figure 7:**
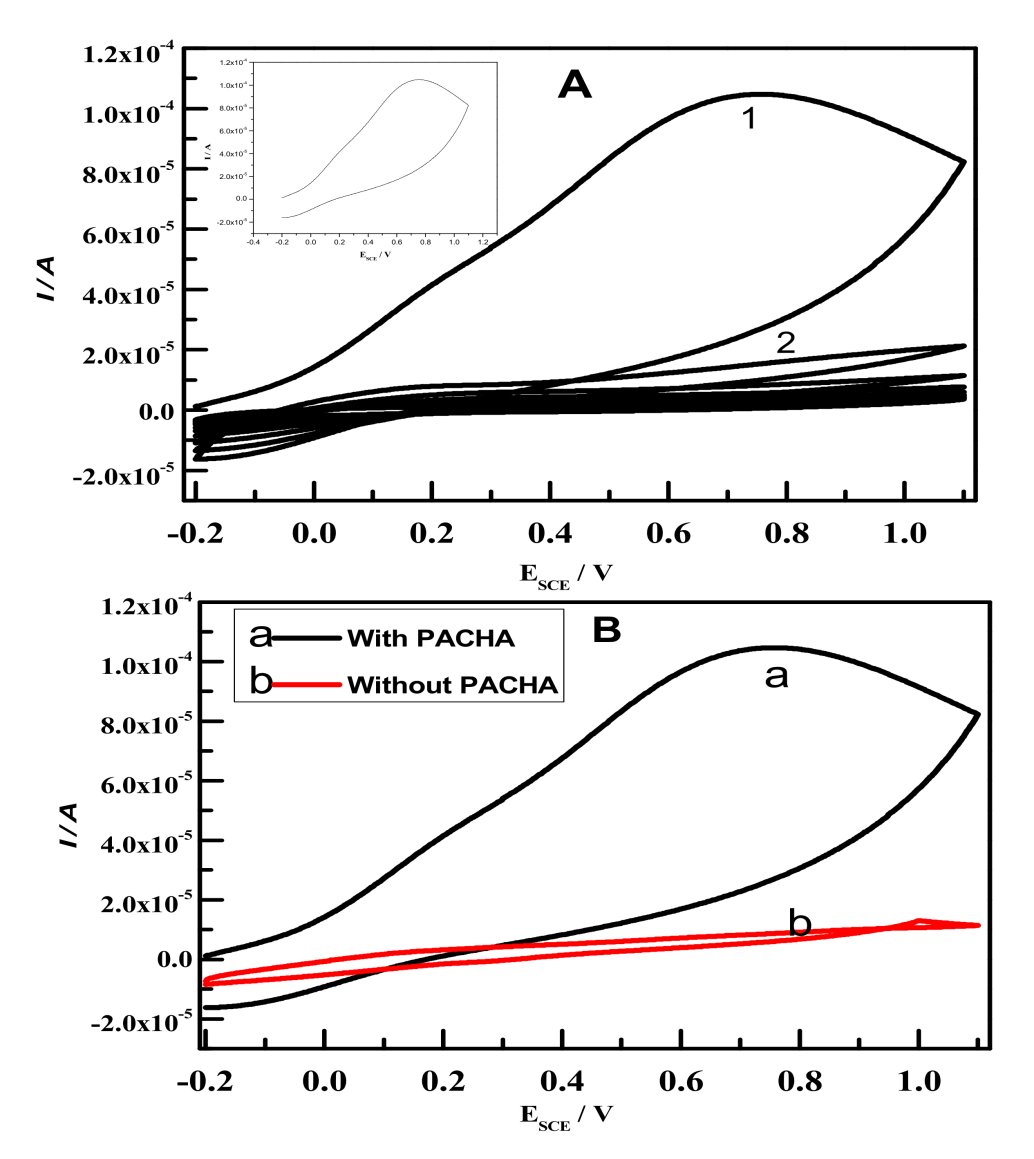
Cyclic voltammograms of (**A**) PACHA coated gold electrode in 0.06 **M** NaOH + **5** M CH_3_OH at 50 mV/s, (the inset showing the first cycle) and (**B**) CVs curves of methanol electro oxidation recorded on PACHA coated and uncoated gold electrodes.

The electro-oxidation of methanol in the high potential region was studied by recording CVs of PACHA coated gold electrodes in 5 **M** CH_3_OH in 0.06 **M** NaOH solution in the potential range of E*_SCE_*= −0.20 V to E*_SCE_*=1.10 V at a scan rate of 50 m V s^−1^ (Figure 7A). During the very first sweep, one broad anodic peak was observed at about 0.74 V with no counterpart in the reverse scan. This peak is attributed to the methanol electro oxidation. In the second cycle, no such peak was observed, which is attributed to the adsorption of intermediates such as CO_ads_ that penetrates into the porous matrix of PACHA coated Au electrodes. The oxides formed in the less positive potential region then further oxidized to form carbonates by losing six electrons according to the following reaction [35]:

CH_3_OH + 8OH^−^ ↔ CO_3_^2−^ + 6H_2_O + 6e^−^ (3)

No such anodic peak was observed in the subsequent potential sweeps though 25 cycles. This is because the intermediates formed covered the surface and no further oxidation took place. Figure 7B shows CVs curves of electro oxidation of methanol on PACHA coated and uncoated gold electrodes. It is obvious that PACHA coated gold substrate enhanced electrocatalytic activity towards methanol oxidation as evidenced by high anodic peak current as compared to the uncoated electrode.

**Figure 8:**
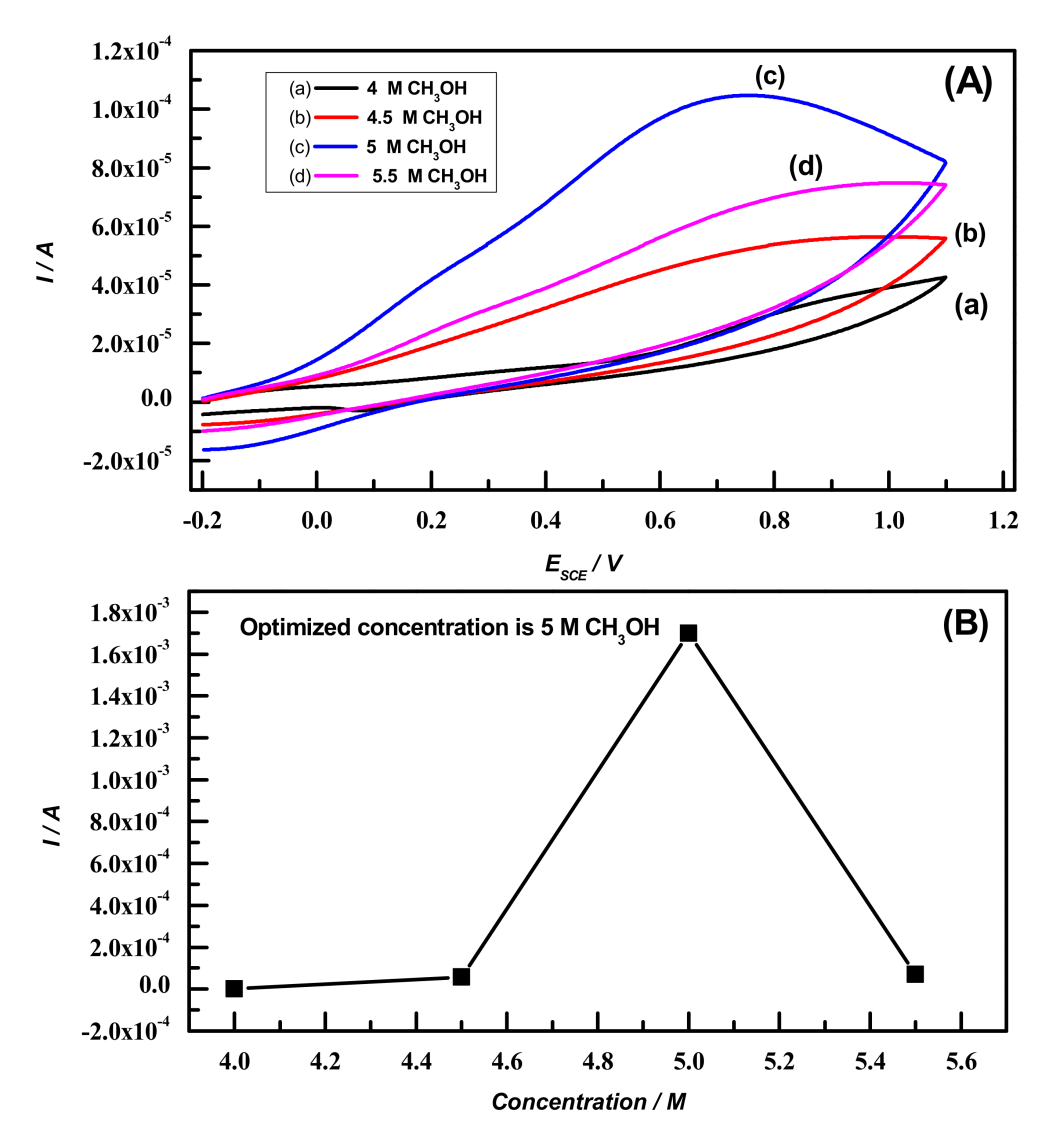
(**A**) CVs of PACHA coated electrodes recoded in different concentrations of methanol (as indicated) in 1.8 M NaOH at 50 mV/s; (**B**) effect of methanol concentration on anodic peak current density in 1.8 M NaOH.

Figure 8A shows the CVs of PACHA coated electrodes recorded in different concentrations of methanol at 50 mV/s. It is clear from the figure that no methanol oxidation takes place in the solution containing 4 **M** CH_3_OH concentration, whereas above 4 **M** concentration a current plateau is observed whose anodic current density increases with an increase in methanol concentration. At 5 **M**, a pronounced broad methanol oxidation peak is observed. Above 5 **M**, a decrease in anodic peak current is observed as depicted in Figure 8B, which means that no more active sites are available for further methanol oxidation [37,66]. On the suggestion of a worthy reviewer, some other concentrations of methanol below and above 5 **M** were also studied, as shown in Figure S4.

**Figure 9:**
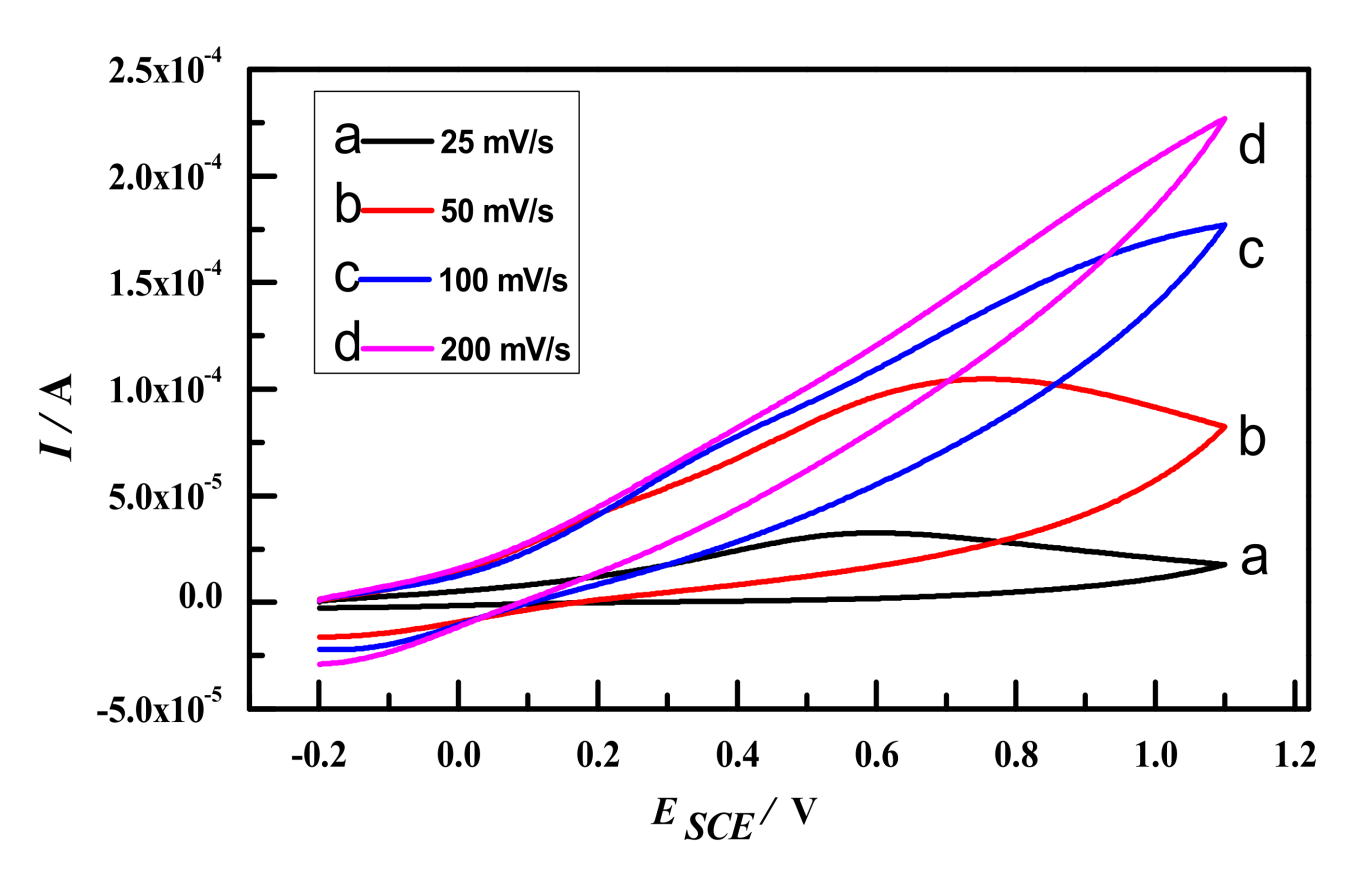
CV curves of PACHA coated gold electrodes in 0.06 **M** NaOH + 5 **M** CH_3_OH at 25, 50, 100, and 200 mV/s.

The effect of scan rate on the anodic peak current of methanol electrooxidation is shown in Figure 9. The anodic peak current at 0.74 V rapidly increases with the increase in sweep rate up to 50 mV/s. Beyond 50 mV/s, no sharp peak is observed around 0.74 V at higher scan rates, indicating near-independence of scan rate. However, a sharp current increase is observed at positive potential limit indicating a faradic type process of methanol electrooxidation.

By observing methanol electrooxidation at both lower and high potential region in different NaOH concentrations, it can be inferred that high pH (1.8 M NaOH) of the supporting electrolyte favors methanol electrooxidation to formats via a facile four electron transfer process. This is supported by the emergence of methanol oxidation peak at less positive potential (value should be added). Under low pH conditions, the oxidation of methanol is kinetically hindered due to the formation of carbonates that is accompanied by six electron transfers. As a result, the oxidation peak appears at high positive potential. In the former case, OH^-^ anion concentration is high and strongly chemisorbed on the surface, which acts as a charge transfer mediator. The formats formed in this case are further oxidized to formic acid as pH of solution is high and there is availability of a greater number of OH^-^ anions. The results clearly indicate that the activity of PACHA coated gold electrodes increases when the pH of solution is high, as already reported elsewhere in literature, supporting our results. PACHA is efficient enough to be used as a membrane in basic fuel cells both at low and high concentrations of the base used.

### 3.3. Electrochemical Impedance Study

**Figure 10:**
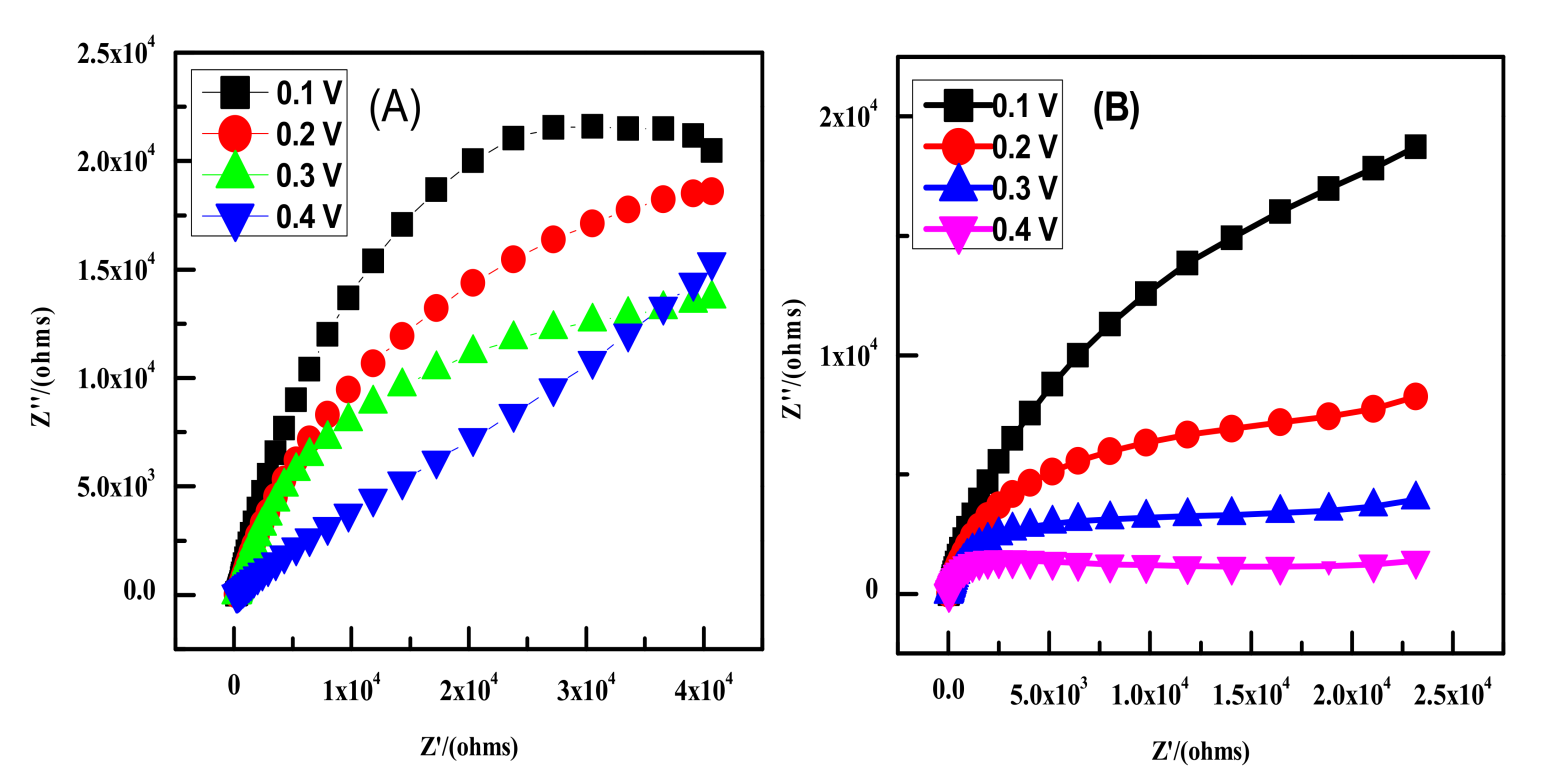
Nyquist plots measured at different potentials as indicated in (**A**) 0.06 **M** NaOH + 5 **M** CH_3_OH and (**B**) in 1.8 **M** NaOH + 5 **M** CH_3_OH solutions.

**Figure 11:**
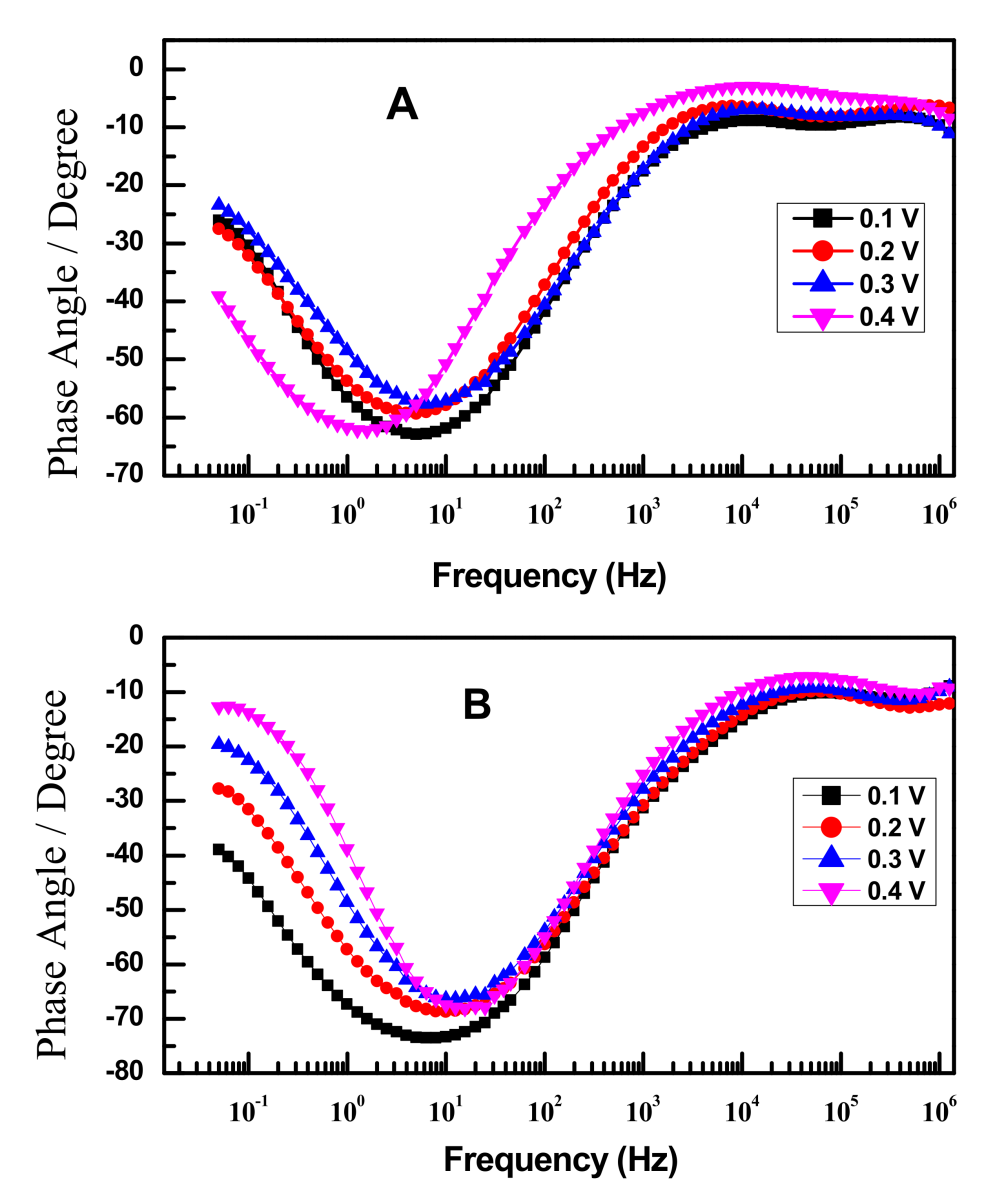
Bode plots measured at different potentials as indicated in (**A**) 0.06 **M** NaOH + 5 **M** CH_3_OH and (**B**) in 1.8 **M** NaOH + 5 **M** CH_3_OH solutions.

The EIS measurements were carried out to investigate interface characteristics of PACHA modified Au electrodes in solutions of both high and low NaOH concentrations with CH_3_OH (5 **M**) at voltages varying from 0.10 V to 0.40 V in the frequency range of 10^−2^–10^6^ Hz. The EIS data were presented both in Nyquist and Bode plots. Figure 10A,B shows the Nyquist plots of PACHA modified Au electrodes in the respective solutions. At lower applied AC voltage, i.e., at 0.1 V, the Nyquist plots in both solutions of high and low concentration of NaOH show semicircles. But after increasing the applied potential, i.e., 0.2–0.4 V, depression in the semicircular arcs are observed. This decrease corresponds to lower charge transfer resistance (R_ct_) as a result of facile kinetics at the electrode-electrolyte interface. The behavior is more pronounced in the case of Figure 10B, where 1.8 M NaOH + 5 M CH_3_OH, i.e., when the solution pH is high. It can also be observed from Bode plots, shown in Figure 11A,B, where the phase degree shift is from −25 to −40 in case of 0.06 M NaOH + 5 M CH_3_OH concentration, whereas when the concentration of base used, i.e., at 1.8 M NaOH + 5 M CH_3_OH solution, the phase degree shift is from −40 to −15 at the low frequency region and shows resistive behavior. CO adsorption occurs during methanol electro oxidation, which poisons the surface catalysis and thus lowers the electrocatalytic activity of the electrode. When the external applied potential is high, the reaction occurring at the anodic surface becomes more pronounce and complete, which results in a decrease in resistance across the interface [67]. The obvious shrinking in the semicircles with the increase in potential indicates decrease in the charge transfer resistance (R_ct_) and corresponding increase in the rate of charge transfer across the electrode/electrolyte interface. Such potential dependent charge transfer behavior was reported during electrooxidation of methanol on carbon nanotube supported high metal content Pt/Ru electrocatalysts [68]. However, no inductive effects, indicated by the existence of inverted semicircles, were observed in the present study. Yang et al. [69] investigated methanol electrooxidation with EIS measurements using Pt particles embedded polyaniline doped with poly(acrylic acid-*co*-malic acid) (PAM) coated electrodes. The Pt surface poisoning due to CO adsorption was greatly hindered by the introduction of PAM into polyaniline. The EIS data presented in our report reveal that PACHA deposited on gold surfaces may also hinder further chemisorption of strongly adsorbed intermediates such as CO on the electrode surface and thus enhances its electrocatalytic activity towards methanol oxidation [70].

**Figure 12:**
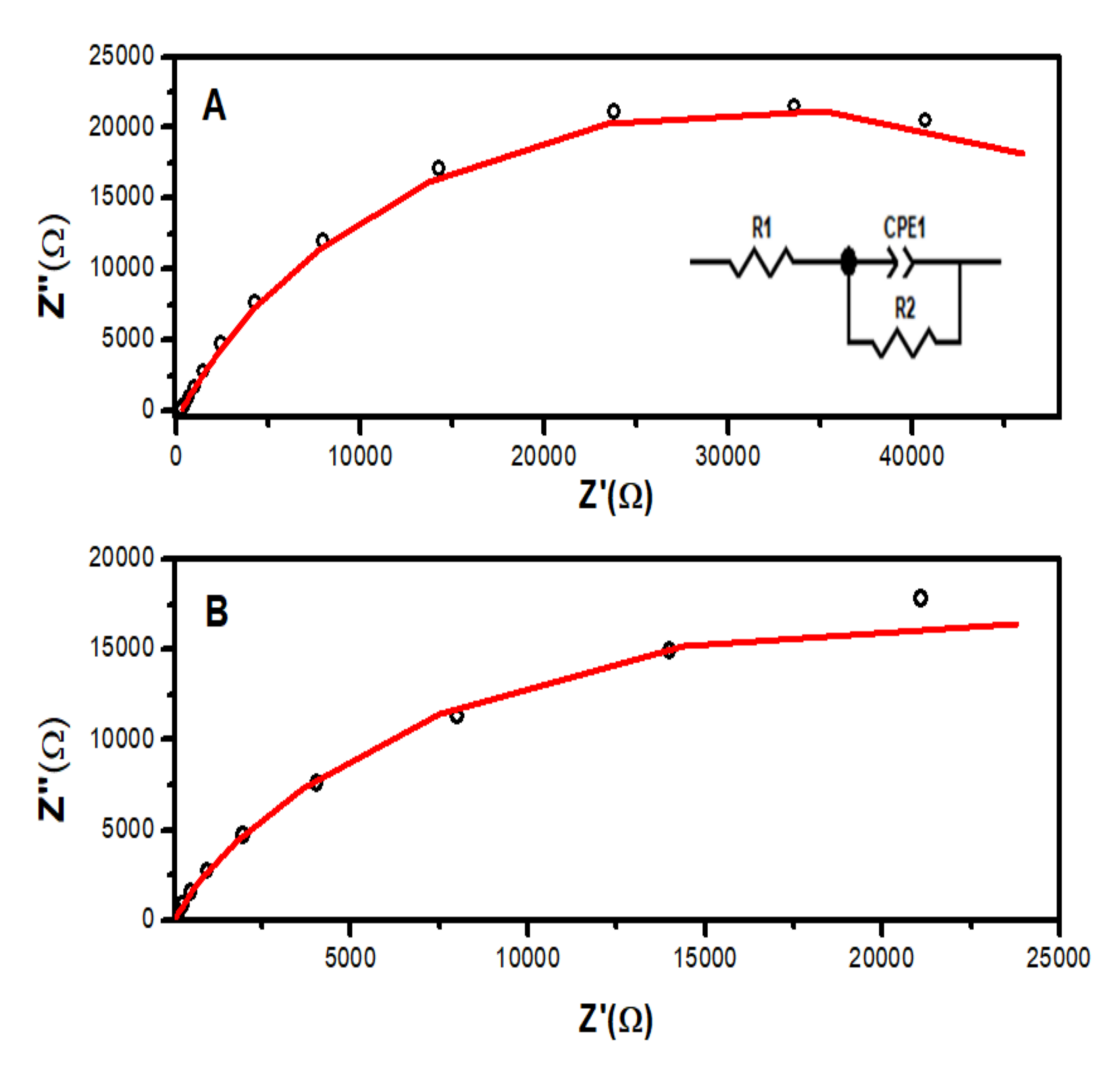
EIS data of PACHA coated gold electrodes at 0.1 V in (**A**) 0.06 M NaOH and (**B**) 1.8 M NaOH in the presence of 5 M CH_3_OH. The dotted data are the experimental results and the solid line is fitted data by an equivalent circuit (inset to Figure 12A).

To obtain quantitative information from the EIS, the results were fitted into equivalent circuits, and the fitted data at 0.4 V are shown in Figure 12. In the circuit, R1 represents the solution resistance, and CPE 1 and R2 are the constant phase element and charge transfer resistance of the electrode and solution interface, respectively. The values obtained from the fitting of data are presented in Table 1. The resistance of 1.8 M NaOH was 31 Ω, which is smaller than that obtained for 0.06 M NaOH (~288 Ω). This is obvious because high concentration of electrolyte conducts more current due to the presence of sufficient ions between the electrodes. Generally, the high constant phase element and low charge transfer resistance values represent better charge transport between electrode and electrolyte solution. Although the CPE value did not change much with high NaOH concentration, which is probably due to the identical nature of electrodes, the charge transfer resistance was found to be greatly reduced in 1.8 M NaOH (~43 kΩ) in comparison to 0.06 M NaOH (~63 kΩ). This indicates the facile methanol oxidation kinetics of PACHA coated gold electrodes in high pH electrolyte, which is consistent with voltammetric results. The properties of electrode materials towards methanol oxidation in basic medium were elsewhere discussed in terms of dielectric constant, capacitances, and AC conductivity without fitting the impedance spectra in an equivalent circuit. However, fitting the impedance data into an equivalent circuit is widely used for interpretation of impedances in terms of charge transfer resistances, constant phase elements, and solution resistances. The fitted data presented in Table 1 clearly indicate fast charge transfer during oxidation of methanol in 1.8 M NaOH as compared to in 0.06 M NaOH solution.

## 4. Conclusions

Conducting polymer based electrode materials are widely used in energy storage and conversion devices. Poly(aniline-*co*-2-hydroxyaniline)(PACHA) was electrochemically synthesized on gold electrodes, characterized with cyclic voltammetry and FTIR spectroscopy, and subsequently applied as an electro-catalyst for oxidation of methanol in basic medium. The behavior of PACHA coated gold electrodes towards methanol electrooxidation was studied at both low and high concentrations of NaOH solution. The PACHA coated electrode significantly enhances the faradaic current of methanol oxidation (20 mA) at comparatively lower potential (30 mV) as compared to uncoated gold substrate (10 mA) in a solution of high base concentration. However, a sluggish response of both coated and uncoated electrodes towards methanol oxidation was found in a solution of low base concentration. The charge transfer resistance for methanol electrooxidation was found to reduce greatly in 1.8 M NaOH (~43 kΩ) in comparison to 0.6 M NaOH (~63 kΩ) at PACHA coated gold electrodes in high pH solution. The facile electrooxidation of methanol on PACHA coated electrodes is observed due to significant reduction in the charge transfer resistance and the hindrance offered by PACHA film for CO adsorption on the gold substrate.

## Figures and Tables

**Table 1:** Equivalent circuit parameters obtained from fitting of the Nyquist plots measured with PACHA coated gold electrodes in different concentrations of NaOH electrolyte containing 5 M CH_3_OH.

Electrolyte	R1 (Ω)	CPE (F)	R2 (Ω)
0.6 M NaOH +5 M CH_3_OH	288	1.63 × 10^−5^	62,755
1.8 M NaOH +5 M CH_3_OH	31.01	4.37 × 10^−5^	43,437

## Data Availability

The data presented in this study are available on request from the corresponding author.

## Supplementary Materials

The following supporting information can be downloaded at: https://www.mdpi.com/article/10.3390/polym14020305/s1, Figure S1. CV curves recorded during oxidation of (a) aniline (b) 2-hydroxyaniline and (c) mixture of aniline and 2-hydroxyaniline; Figure S2. CV curves recorded during oxidation of electropolymerization of aniline. The inset show CV of PANI coated electrode in monomer free electrolyte solution; Figure S3. Anodic peak current vs square root of sweep rate; Figure S4. CVs recorded during electrodeposition of PACHA on gold electrode during electrolysis of a solution containing containing 1m**M** 2-hydroxyaniline and 20 m**M** aniline in 0.5 **M** sulphuric acid solution at 50 mV/s; Figure S5. CVs of PACHA coated electrode recoded in different concentrations of methanol (as indicated) in 1.8 M NaOH at 50 mV/s.
