# Supplementary material for: Performance Improvement of Gold Electrode towards Methanol Electrooxidation in Akaline Medium: Enhanced Current Density Achieved with Poly(aniline-co-2-hydroxyaniline) Coating at Low Overpotential
Source: Polymers (Basel). 2022 Jan 13;14(2):305. doi: 10.3390/polym14020305 (PMC8780982; doi:10.3390/polym14020305)
Supplement: Supplementary file 1 [file polymers-14-00305-s001.zip › polymers-1524235-supplementary.pdf]

Supporting information

Performance Improvement of Gold Electrode towards Methanol Electrooxidation in Akaline Medium: Enhanced Current Density achieved with Poly (aniline-co-2-hydroxyaniline) Coating at Low Overpotential

Anwar ul Haq Ali Shah [1,*] , Sadaf Zia [1], Gul Rahman[1], Salma Bilal [2]

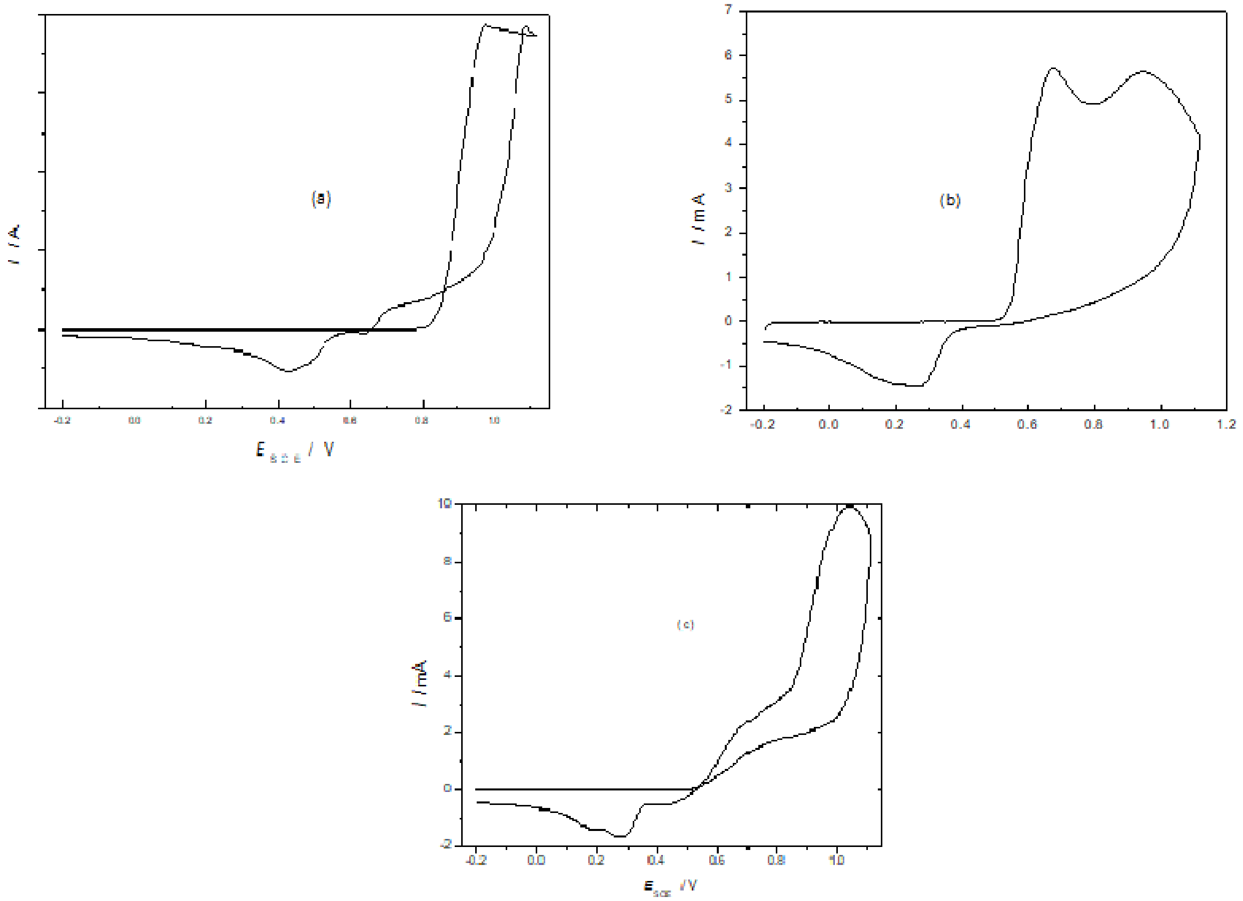

Figure S1. CV curves recorded during oxidation of (a) aniline (b) 2-hydroxyaniline and (c) mixture of aniline and 2-hydroxyaniline.

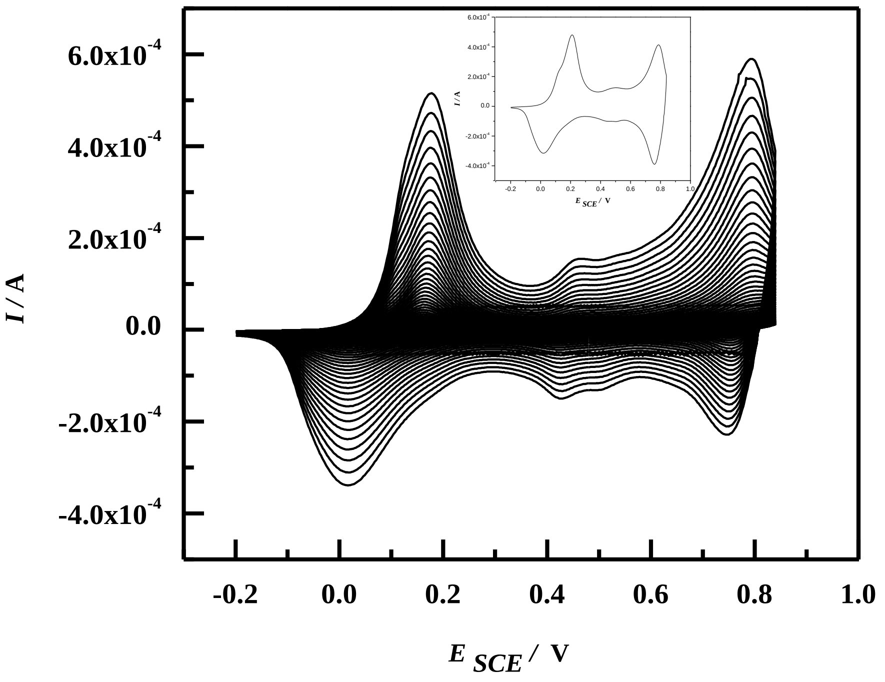

Figure S2. CV curves recorded during oxidation of electropolymerization of aniline. The inset show CV of PANI coated electrode in monomer free electrolyte solution.

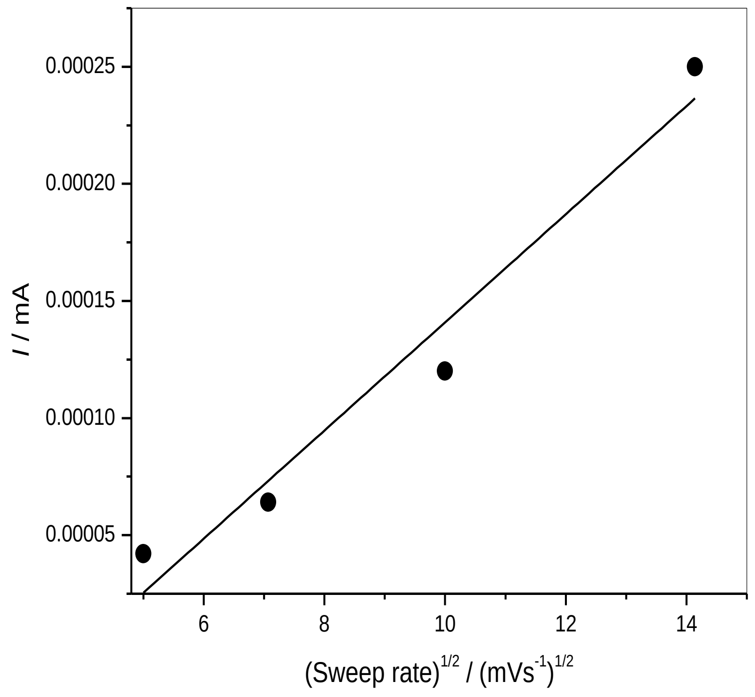

Figure S3. Anodic peak current vs square root of sweep rate.

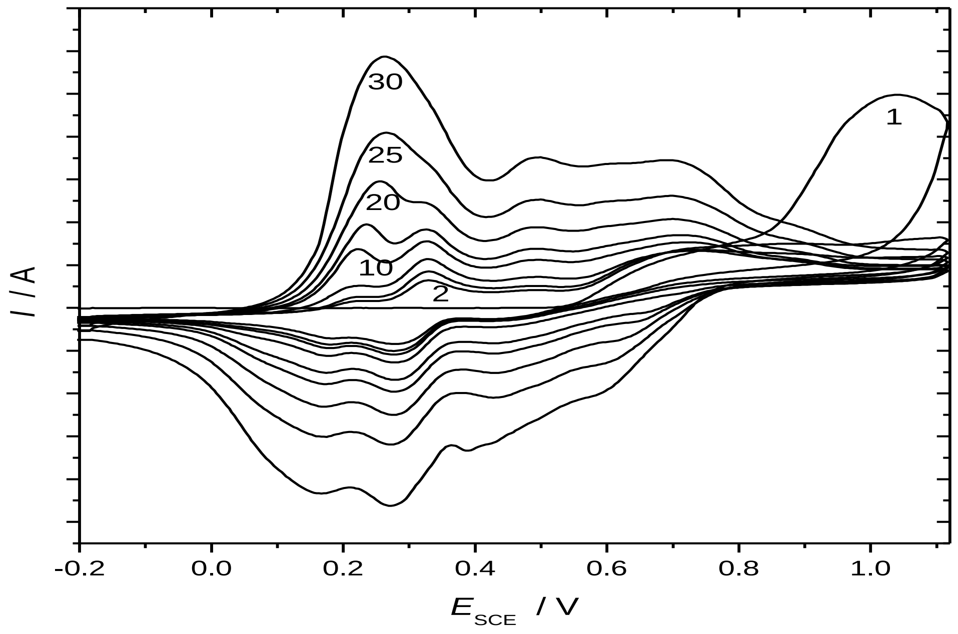

Figure S4. CVs recorded during electrodeposition of PACHA on gold electrode during electrolysis of a solution containing containing 1m**M** 2-hydroxyaniline and 20 m**M** aniline in 0.5 **M** sulphuric acid solution at 50 mV/s.

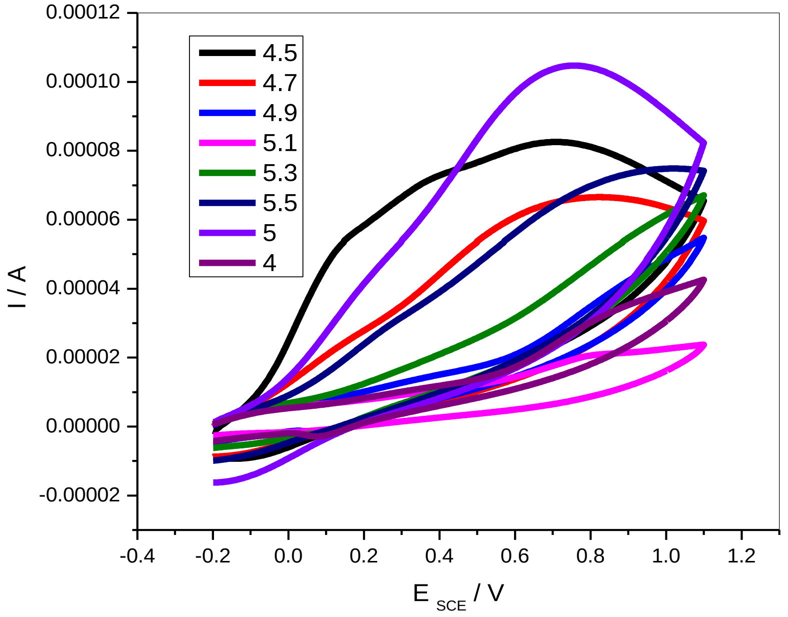

Figure S5. CVs of PACHA coated electrode recoded in different concentrations of methanol (as indicated) in 1.8 M NaOH at 50 mV/s.
